# HOW CAREGIVING SHAPES THE RELATIONSHIP BETWEEN ACTIVITY DIVERSITY AND COGNITION

**DOI:** 10.1093/geroni/igae098.3663

**Published:** 2024-12-31

**Authors:** Cassidy Doyle, Brent Small

**Affiliations:** University of South Florida, Tampa, Florida, United States; University of North Carolina at Chapel Hill, Chapel Hill, North Carolina, United States

## Abstract

Research indicates frequent participation in leisure activities may buffer age-related cognitive declines but activity variety (activity diversity) may have a stronger relationship with cognitive health. The activity diversity-cognition relationship is understudied in caregivers, who may be susceptible to cognitive declines and therefore benefit from leisure activity participation. This study examines cognitive differences between caregivers and non-caregivers and explores the roles of activity diversity and frequency in this relationship. The Health and Retirement Study was used to investigate the relationship between activity diversity, activity frequency, and cognition among caregivers (N=6,472) and non-caregivers (N=4,343). From the Psychosocial and Lifestyle Questionnaire, 17 leisure activities were assed to create activity diversity and frequency measures. Activity diversity was quantified using Shannon’s entropy, scores ranging from 0 to 1, with higher scores indicating greater diversity. Activity frequency was measured by summing the frequencies of 17 activities (0=not at all to 5=daily). Moderation analysis assessed the relationships between activity diversity-cognition and activity frequency-cognition by caregiving status. The sample was predominantly female, White, educated, and engaged in diverse activities (M=0.49). Caregivers had lower cognition than non-caregivers (p <.001). Moderation analyses showed that higher activity diversity and frequency were associated with better cognition for both groups, with stronger effects for non-caregivers (p <.001). Greater leisure frequency and diversity were related to better cognition. Unexpectedly, caregivers engaged in more frequent and diverse leisure compared to caregivers, but associations between activity engagement-cognition were stronger in non-caregivers. Investigating the potential for activity engagement to benefit multiple domains (wellbeing, stress, cognition) among caregivers is warranted.

